# The Stanford Medicine data science ecosystem for clinical and translational research

**DOI:** 10.1093/jamiaopen/ooad054

**Published:** 2023-08-02

**Authors:** Alison Callahan, Euan Ashley, Somalee Datta, Priyamvada Desai, Todd A Ferris, Jason A Fries, Michael Halaas, Curtis P Langlotz, Sean Mackey, José D Posada, Michael A Pfeffer, Nigam H Shah

**Affiliations:** Stanford Center for Biomedical Informatics Research, Stanford University, Stanford, California, USA; Department of Medicine, School of Medicine, Stanford University, Stanford, California, USA; Department of Genetics, School of Medicine, Stanford University, Stanford, California, USA; Department of Biomedical Data Science, School of Medicine, Stanford University, Stanford, California, USA; Technology and Digital Solutions, Stanford Medicine, Stanford University, Stanford, California, USA; Technology and Digital Solutions, Stanford Medicine, Stanford University, Stanford, California, USA; Technology and Digital Solutions, Stanford Medicine, Stanford University, Stanford, California, USA; Stanford Center for Biomedical Informatics Research, Stanford University, Stanford, California, USA; Technology and Digital Solutions, Stanford Medicine, Stanford University, Stanford, California, USA; Department of Radiology, School of Medicine, Stanford University, Stanford, California, USA; Department of Anesthesia, School of Medicine, Stanford University, Stanford, California, USA; Technology and Digital Solutions, Stanford Medicine, Stanford University, Stanford, California, USA; Technology and Digital Solutions, Stanford Medicine, Stanford University, Stanford, California, USA; Stanford Center for Biomedical Informatics Research, Stanford University, Stanford, California, USA; Technology and Digital Solutions, Stanford Medicine, Stanford University, Stanford, California, USA; Clinical Excellence Research Center, School of Medicine, Stanford University, Stanford, California, USA

**Keywords:** patient data repositories, electronic medical records, data science, team science, informatics

## Abstract

**Objective:**

To describe the infrastructure, tools, and services developed at Stanford Medicine to maintain its data science ecosystem and research patient data repository for clinical and translational research.

**Materials and Methods:**

The data science ecosystem, dubbed the Stanford Data Science Resources (SDSR), includes infrastructure and tools to create, search, retrieve, and analyze patient data, as well as services for data deidentification, linkage, and processing to extract high-value information from healthcare IT systems. Data are made available via self-service and concierge access, on HIPAA compliant secure computing infrastructure supported by in-depth user training.

**Results:**

The Stanford Medicine Research Data Repository (STARR) functions as the SDSR data integration point, and includes electronic medical records, clinical images, text, bedside monitoring data and HL7 messages. SDSR tools include tools for electronic phenotyping, cohort building, and a search engine for patient timelines. The SDSR supports patient data collection, reproducible research, and teaching using healthcare data, and facilitates industry collaborations and large-scale observational studies.

**Discussion:**

Research patient data repositories and their underlying data science infrastructure are essential to realizing a learning health system and advancing the mission of academic medical centers. Challenges to maintaining the SDSR include ensuring sufficient financial support while providing researchers and clinicians with maximal access to data and digital infrastructure, balancing tool development with user training, and supporting the diverse needs of users.

**Conclusion:**

Our experience maintaining the SDSR offers a case study for academic medical centers developing data science and research informatics infrastructure.

## OBJECTIVE

The objective of this work is to describe the Stanford Medicine Data Science Resources (SDSR) which comprise the Stanford Medicine Research Data Repository (STARR) and the infrastructure and services developed at Stanford Medicine to maintain and provide access to STARR for clinical and translational research.

## BACKGROUND AND SIGNIFICANCE

A typical health system has hundreds of independent information technology (IT) systems, each capturing specific data modalities over time, at widely varying levels of granularity and frequency, and for different purposes. Research patient data repositories at academic medical centers (AMCs)^1–8^ provision these data to advance data science and artificial intelligence (AI) applications in medicine,^9^ including the design and evaluation of machine learning models to identify and predict clinical events,^10^^,^^11^ to enable clinical trial recruitment and prospective clinical research,^12^ and to serve as sources of data for large scale observational studies.^13–15^ To be useful for research, operational and transactional data must be transformed into events on a patient timeline that aggregates data from multiple sources (imaging studies, bedside monitors, electronic medical records, pharmacy records, insurance claims), to provide a comprehensive record of each patient’s interactions with the health system, and their outcomes over time.^16^ Research data repositories must also balance providing “processed” data with providing raw data that are amenable to custom analysis, such as information extraction from unstructured clinical text.^17–20^ Achieving this balance is an active process requiring engagement of a broad community of stakeholders.

We have built on the foundation of the Stanford Translational Research Integrated Database Environment (STRIDE),^21^ evolving it into the STARR to link resources comprising the SDSR.^19^^,^^22^ This process began with the conception and design of STARR in 2016, which expanded to new data sources, tools, and infrastructure in 2017–2021. In this paper, we describe the infrastructure, tools, and services developed during this evolution, as well as the teaching, clinical and translational research, and collaborations it enables. We also compare the SDSR to data science platforms developed at other Clinical and Translational Science Award (CTSA) Program Hubs.

## MATERIALS AND METHODS

The SDSR comprises compute and data infrastructure, services for data deidentification, linkage, and processing to extract information from different healthcare IT systems, and tools to create, search, retrieve, and analyze patient data. Linked patient data generated by these services are stored using a common data model maintained by the Observational Health Data Sciences and Informatics (OHDSI) community. Data are made available in deidentified form via self-service as well as concierge supported access, on HIPAA compliant secure computing infrastructure, with the ability to link to images, waveforms, and wearables data. The SDSR is maintained via coordination amongst approximately 30 engineers and analysts as well as 10 honest broker personnel in Stanford Medicine’s Technology and Digital Solutions (TDS) team,^23^ Research Informatics Center (RIC), and the Stanford Research Computing Center (SRCC).^24^ Each SDSR component is described in the following dedicated sections, with references to technical whitepapers for additional details.

### Infrastructure

#### A common data model to organize clinical data

STARR^19^ uses the OHDSI Observational Medical Outcomes Partnership (OMOP) Common Data Model (CDM),^25^ which provides interoperability across research centers and data sources^25–27^ to enable OHDSI network studies.^14^ The OMOP CDM is used widely by a large community of developers and researchers^26^^,^^28^^,^^29^ to support a suite of open source data processing and analysis tools in the OHDSI community for creating cohort definitions, analysis designs, and reporting of results.^30^

The OMOP CDM captures patient-specific variables including demographics, diagnosis records, procedure records, medication records, physiologic measurements (vital signs, height, weight etc.), laboratory test results, structured content extracted from clinical notes via text processing, as well as information about providers and health systems. The OMOP CDM does not yet provide a data representation scheme for other data such as images but related efforts are in progress, including a pilot implementation of Picture Archiving and Communication System (PACS) data in the OMOP CDM.^31^

#### Computing resources

We use Google Cloud Platform (GCP) under a Business Associate Agreement (BAA) between Stanford and Google. We use containerization solutions for software encapsulation, including Docker and Singularity. We use GCP to instantiate virtual machines for data processing (described below) and provisioning data to researchers as BigQuery datasets. The decision to use cloud infrastructure is based on our experience in developing data processing workflows in genomics, where cloud versus on-premise infrastructure, including data center support staff, did not differ substantially in terms of cost.^32^ However, cloud computing offers the ability to easily experiment with new software stacks, eg, comparing tools such as DBT^33^ and WDL,^34^ as well as technical advantages including faster query times using BigQuery, the ability to scale up computational resources on-demand, and the ability to instantiate data science toolkits for researchers *within* a secure environment.^35^

#### Self-serve access to secure computing infrastructure and deidentified data

SRCC maintains a secure data science platform, Nero, which uses a combination of on-premise servers, containerization, and cloud computing to support large scale data analytics^36^ by providing researchers with self-serve access to compute environments with tools such as Jupyter notebooks, Python, Anaconda, TensorFlow, and RStudio. Nero also supports OHDSI tools including the ATLAS tool for search and cohort building and underlying R packages. TDS maintains researcher self-service cohort building and tools developed internally at Stanford,^21^ and the OHDSI ATLAS web-based cohort analysis tool, for creating cohort definitions and building patient datasets from STARR data. Costs for using Nero vary with usage, but on average users can expect to spend ∼$40/month/TB for storage, ∼$25/month/TB (storage) and ∼$5/TB (query) for BigQuery, and ∼$100/month for running a compute instance with 30GB RAM, a 100 GB disk, and 8vCPUs.^37^

Stanford University classifies even deidentified patient data as high risk, and thus all SDSR datasets (see “Results”) are provisioned via Nero. To gain access to deidentified data on Nero, researchers complete privacy training and sign a data use agreement, referred to as a Data Privacy Attestation, that prohibits recipients from attempting to reidentify the data subjects, or from sharing the data. The signed Data Privacy Attestation serves as a record of who has access to the deidentified data, and their agreement to its terms of use. Researchers who access high risk data are also required to use laptops configured with encrypted hard drives by University IT, and to attest that they only store high risk data on approved compute environments.

### Services

#### Data ingestion and quality assurance

The Stanford adult and children’s hospitals are on separate Epic instances and corresponding Clarity data warehouses. When patients check in for an appointment at either of the hospitals they provide informed consent regarding how their data (identified or deidentified) may be collected by these systems and used in providing care or for research. SDSR services migrate data from Clarity to GCP BigQuery using the Apache Avro format on a weekly basis^19^ for extract-transform-load (ETL) into STARR.

Two essential and related components of the ETL are: (1) deidentification and (2) patient identifier creation and maintenance. The deidentification process first removes structured fields containing known identifying information (Medical Record Numbers, Social Security Numbers, names, addresses etc.), and also deidentifies unstructured data including notes and images (as described in the “Deidentification of Notes and Images” section below). A randomly chosen date shift is then applied to the dates of each patient record. The same date shift value is used for all records associated with a single patient such that the amount of time between events for each patient remains unchanged. Lastly, a new random “person identifier” is created for each patient, which is consistent across STARR datasets to enable automatic linkage. In other words, a query for Person “12345” will retrieve all records across STARR datasets for the same patient. Identifiers are also persistent across data refreshes, such that a query for Person “12345” to a dataset created in Q1 2021 and an updated version of that dataset created in Q2 2021 will retrieve data for the same patient. We also maintain “codebooks” that keep track of patient medical record numbers (MRNs) from Epic Clarity and their corresponding person identifiers created for each patient during ETL in a secure location with access restricted to TDS staff. The date shift applied for each patient is also stored in the patient identifier codebooks. These codebooks enable linkage from STARR deidentified datasets to other project-specific datasets that may contain identified data (eg, data collected by providers as part of IRB approved human subjects research) without releasing identifying information to investigators.

The ETL also balances providing processed data with providing raw data that are needed in original form for research purposes. For example, the ETL converts partially unstructured data from clinical flowsheets into structured records, such as vital sign measurements including blood pressure, oxygen level, heart rate, respiratory rate, Sequential Organ Failure Assessment (SOFA) scores, Glasgow Coma Scale Scores, and Deterioration Index scores, but ingests and provides access to clinical notes as-is. The ETL preserves visit-level linkage of encounter details (such as diagnoses, procedures etc.) from the source Clarity data.

During and after ETL, we use a combination of custom processes and OHDSI tools including the Data Quality Dashboard (DQD) for data quality checks. Internal quality assurance processes include manual review of small numbers of records to ensure integrity across source records and their OMOP CDM counterparts, and comparison of aggregate counts of clinical events over time to identify anomalous variation that could indicate errors in ETL.

In addition to data from Epic EMRs, the SDSR also hosts radiology, cardiology, and bedside monitor data. In 2018, we ingested all historical radiology data from the shared Stanford Health Care and Stanford Children’s Health PACS into STARR. In 2021, we redesigned the imaging ingestion pipeline to an incremental model that pushes PACS data updates on a daily basis to STARR from a Vendor Neutral Archive (VNA) that aggregates imaging data from multiple clinics and applications.^19^^,^^38^ This redesign, part of the larger evolution of STRIDE into STARR, has eliminated the need for large, expensive retrieval, and deidentification requests from Stanford’s PACS. The processing, cleaning, and deidentification rely on the DICOM standard^38^ and are independent of the ingestion mechanism. The DICOM processing pipeline supports both radiology and cardiology DICOM records.

Bedside monitoring data include waveform and vitals signs from patient monitors, telemetry devices, and third-party devices connected to the Philips IntelliBridge family, such as heart rate, blood pressure, pulse oximetry, alarms and alerts, and continuous waveforms such as electrocardiograms and invasive pressures. A nightly extract is compressed, deidentified, and copied to GCP cloud storage. Data is validated at this stage by verifying daily counts in the clinical database against the number of rows in the extracted files. Identified and deidentified vitals and metadata are stored in separate cloud storage locations and datasets. We also generate metadata to record if a given list of patients, bed locations or calendar times have bedside monitoring data and store the locations of the corresponding data, enabling researchers to identify waveform and vitals records specific to patients or studies of interest. The data are cross-linked with EMRs as well as other sources such as electroencephalogram (EEG), radiology, and video monitoring data.^39^ We use Google BigQuery for storing and retrieving metadata and cloud storage for storing and retrieving raw and waveform data. We also use on-demand GCP virtual machines for the data processing described above.

#### Deidentification of notes and images

In addition to the deidentification of structured patient data, we use a 2-step process to deidentify clinical text. We first use TiDE, a hybrid NLP approach composed of CoreNLP^38^^,^^40^ and pattern-matching heuristics, to find mentions of identifying information. We then use a “hiding in plain sight” (HIPS)^41^ approach, whereby we replace identifiers (including names, places, and addresses) flagged by TiDE with surrogate text. For example, names are detected using a database of known names from source data and are replaced with surrogate names. If TiDE misses a mention of a real name, using HIPS ensures that it will not be apparent which names are original and which have been replaced. At the time of writing, name replacement is gender aware but not ethnicity aware. Surrogate addresses are selected randomly. The results of deidentification are reviewed via manual quality control, described in Datta et al, supplement 6.^19^

To deidentify clinical images we developed a distributed software application that operates on-demand in response to user requests for images.^38^ The deidentification mechanisms are based on the Radiological Society of North America Clinical Trial Processor (CTP)^42^ updated with custom filtering, deidentification, and pixel scrubbing rules to manage Stanford-specific imaging types and features.^38^ This on-demand service avoids having to deidentify images in large batches (Stanford Medicine generates ∼450 terabytes of radiology imaging data each year), enabling us to meet researcher needs while maintaining computational efficiency.

#### Entity extraction from text

SDSR services process clinical notes using an entity recognition pipeline^43^ to provide researchers with a simple representation of provider note content. This pipeline constructs a dictionary of clinical terms from the Unified Medical Language System terminologies^44^ and searches for mentions of these dictionary terms in all provider notes. Using additional rule-based modules based on note section headers, as well as negation^45^ and context^46^ detection methods, we flag each mention to indicate whether it is negated, about the patient (vs a family member, as in the Family History section of notes) and present or past tense. The processed output only retains whether a term occurred in a given note or not. This “bag-of-words” representation of the content of clinical notes can then be used for advanced electronic phenotyping^47–50^ alongside other structured data such as diagnosis codes, procedure records, vitals, and laboratory test results.

### Governance and funding

The priorities and activities of the TDS, RIC, and SRCC teams are informed by 2 advisory committees—the Research Technology Advisory Committee and the Dean’s Office Governance committee—composed of School of Medicine faculty, the Office of the Senior Associate Dean of Research, and Stanford Health Care leadership including the Chief Information Officer and Chief Analytics Officer. The first provides advice via a transparent, consensus driven process for investments and management of technology that supports our research mission. The second synthesizes input from other advisory committees (such as on Education Technology and Administrative IT systems) to make a recommendation for approval by the Dean’s office. This approach is analogous to NIH grant reviews where a study section scores a proposal on its scientific merits, and then the relevant institute’s program office and council score based on alignment with strategic priorities. An independent Data Management and Access committee decides on which data can be used for what purpose as well as manages decisions around requests to access financial or other sensitive data for research. Prioritization is also informed by user requests, for example if many STARR users request a specific data type or tool functionality, resources are dedicated to that work.

The personnel, services, and datasets that make up the SDSR are supported with a mix of institutional and grant sources. Institutional support includes funding from the School of Medicine Dean’s Office, Stanford’s CTSA (Spectrum), and Stanford Health Care (as the parent organization of the TDS team). Investigators that use SDSR resources, including BigQuery and GCP infrastructure, allocate grant funding to support their use. Concierge Service consultations (described above) are subsidized by the Dean’s Office; any resulting custom data extracts may require additional support from other sources, such as investigator grants, to cover compute infrastructure and labor. In the future, SDSR will implement a cost recovery model for use of STARR datasets and imaging deidentification, whereby investigators and TDS jointly create Statements of Work to be invoiced and reimbursed from investigator funds. In general, Dean’s Office funds support new SDSR projects and infrastructure improvements (eg, the addition of a new data source to STARR, such as whole-slide pathology data), and maintenance is supported via cost recovery.

## RESULTS

**Figure 1.**
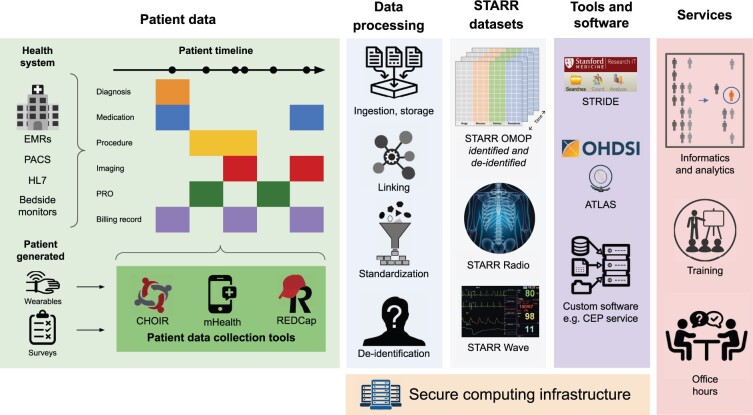
Overview of the SDSR ecosystem. From left to right: the sources of data that comprise patient timelines, which are processed to create the STARR datasets that can be retrieved and analyzed using community and internally developed tools. These processing systems, datasets and tools are maintained on a secure computing infrastructure. Consulting support in the form of informatics and analytics services, user training, and office hours, is provided.

The STARR was launched in the fall of 2019, and includes EMR data, deidentified clinical images and text, bedside monitor data, and HL7 messages. STARR serves as an integration point for other SDSR components, including a real-time alerting system for clinical trials recruitment and tools for collecting patient reported outcomes, survey responses, and data from wearables (Figure 1). This collection of resources anchors the development of analytic tools, supports reproducible research, enhances graduate teaching on the use of healthcare data, and enables industry collaborations and international clinical studies.

### STARR datasets

STARR houses patient-level data from a number of sources (see “Materials and Methods” section titled “Data Ingestion and Quality Assurance”) consisting of many data types, each of which we describe below. Datasets in STARR (Table 1) can be cross-linked on a project-specific basis with other datasets.

**Table 1.** Year range, number of records, and approximate size of each STARR dataset

Dataset	Year range	Number of records (unit)	Size (TB)
STARR OMOP	1997–present	>3 400 000 (patients)	0.3
STARR Radio	2010–present	>1 000 000 000 (images)	2000
STARR Wave	2017–present	>1 000 000 (studies)	17

*STARR OMOP* ^51^ is the primary clinical data warehouse for Stanford Medicine researchers, with records for more than 3.4 million patients from the adult hospital and outpatient clinics that make up Stanford Health Care and the Lucile Packard Children’s Hospital and its affiliated clinics that comprise Stanford Children’s Health. STARR OMOP data are updated and released monthly to the research community. Hundred percent of the patients with at least one encounter in STARR OMOP have age and date of birth on record. Sixty-four percent of patients have at least one diagnosis code, over 46% have medication information, 78% have laboratory test information, and over 85% of patients have clinical notes data available. STARR OMOP data are available to researchers in both identified and deidentified form, as described above.

*STARR Radio* ^38^ contains records from the Stanford PACS, from over 5.7 million studies and over 1 billion Digital Imaging and Communications in Medicine (DICOM) records, totaling more than 2 petabytes of data. This represents over 95% of the data in PACS generated over the last 10 years. STARR Radio data includes images from multiple modalities such as radiography, computed tomography (CT), magnetic resonance imaging (MRI), positron emission tomography (PET), and ultrasound (including video), from both the adult and children’s hospitals.

*STARR Wave* ^39^ ^,^ ^52^ consists of pediatric bedside monitoring data linkable to EMR data in STARR OMOP and radiology data in STARR Radio. Bedside monitors capture heart rate, blood pressure, pulse oximetry, and electrocardiogram data at second-level resolution, as well as alerts related to these vital signs. These data include an average of 180 000 alerts from hundreds of monitors for approximately 280 patients per day, corresponding to approximately 75 gigabytes of data extracted per day and a total of more than 17 terabytes as of this writing. Future versions of STARR Wave will include data from the adult hospital as well as the children’s hospital.

### SDSR tools

A key component of the SDSR ecosystem is the development and maintenance of tools to support clinical data science. Table 2 summarizes tools developed at Stanford and available to Stanford Medicine researchers for patient data collection, analytics, and machine learning for diverse informatics and research applications. These tools operate over the foundation of the data ingestion, processing (including deidentification), and access mechanisms described in the “Materials and Methods” section.

**Table 2.** Tools for data collection, analytics, and machine learning in the SDSR ecosystem

Tool	Summary	Publications
**Patient data collection**		
CHOIR	The Collaborative Health Outcomes Information Registry (CHOIR) is an open-source platform for generating, collecting, and summarizing patient-centered data via integration with EMR software using SMART on FHIR, delivering surveys and tracking patient responses. CHOIR data can be directly linked to STARR datasets.	^53^ ^,^ ^54^
mHealth	mHealth comprises HIPAA-compliant services that provide secure storage and processing infrastructure for data collected via mobile devices such as smartphones and watches. mHealth services are not restricted to Stanford Health Care patients, and thus cannot be directly linked to STARR datasets.	^55–59^
REDCap	REDCap is a web platform that allows researchers and clinicians to develop surveys, securely collect participant data and export that data for analysis. REDCap Surveys are not restricted to Stanford Health Care patients, and thus cannot be directly linked to STARR datasets.	^60–63^
CEP Engine	The Complex Event Processing (CEP) engine processes the live Stanford Health Care HL7 data feed to identify patients who may be eligible for clinical trials operating out of Stanford Medicine. CEP alert data can be linked to STARR datasets upon request.	^64^ ^,^ ^65^
**Data analytics**		
ACE	The Advanced Cohort Engine (ACE) is a scalable patient search engine and datastore designed for electronic phenotyping and building patient cohorts. ACE is deployed over STARR OMOP data.	^66^
ePAD	The electronic Physician Annotation Device (ePAD) is a web-based imaging informatics platform for quantitative imaging analysis. ePAD data can be linked to STARR datasets upon request.	^67^
**Machine learning**		
Trove	A natural language processing (NLP) framework using weak supervision for named entity recognition (NER), attribute classification, and relation extraction. Trove operates over clinical notes available in STARR OMOP.	^20^
CLMBR	Clinical language model-based representations (CLMBR) is a deep learning architecture for learning transferable feature representations of patient timelines, enabling the development of patient classifiers for risk stratification and time-to-event models. CLMBR operates over patient records in STARR OMOP.	^68^

SDSR data migration and data deidentification services have also enabled the public release of a large number of data sets, including 19 imaging data sets spanning a variety of imaging modalities and body systems and comprising hundreds of thousands of imaging studies and millions of images.^69–72^ These large high-quality clinical datasets are available to the broader research community beyond Stanford. The majority of these data sets has been compiled from Stanford Health Care patient records and is provided specifically for the purpose of developing and validating machine learning applications.

### SDSR user community

SDSR supports more than 120 faculty research groups, more than 800 cloud compute users, and more than 300 self-serve deidentified STARR data users as of this writing. Since March 2020, the number of STARR data users has increased by 86% and the number of principal investigators using STARR data and services by 70%. As a baseline for comparison, approximately 630 users searched patient data using STRIDE tools (the other primary entry point for Stanford patient data access) in 2021, with more than 460 conducting chart review. Approximately 25–30 investigators per quarter receive concierge support by RIC for data delivery and analysis.

### Research and education

TDS provides workshops, office hours, and documentation as an integral part of the SDSR ecosystem. Day-long tutorials train users in data science tools and resources for analyzing STARR datasets, as well as provide the TDS staff a view into researchers’ processes and methodology. The tutorial series “Stanford Medicine Tools for Healthcare Data Science” is available as a YouTube channel.^73^ We also maintain a Gitlab with sample code.^74^ To date, we have trained approximately 115 researchers in our workshops and held more than 125 office hours.

The Biomedical Informatics graduate course, *Data Science in Medicine*, teaches students to search, retrieve, process, and analyze deidentified patient data from STARR OMOP via lectures and projects. Similarly, the Epidemiology undergraduate and graduate course *Big Data Methods for Behavioral, Social, and Population Health Research* incorporates hands-on experience using OHDSI tools with STARR deidentified patient data. The Biodesign course *Biodesign for Digital Health* gives students the opportunity to prototype tools using resources such as the mHealth platform to tackle real digital health challenges. In all of these courses, students gain experience working with real clinical data and tools, with minimal overhead for instructors, while protecting patient privacy via SDSR deidentification and access control services.

The combination of data, tools, training, and course offerings have enabled diverse research including hackathons and challenges,^75–79^ multi-institutional^80–87^ and industry collaborations,^88–90^ interdisciplinary studies,^91^^,^^92^ and health system implementations of informatics-driven research.^93–96^

### Comparison to data science platforms at other CTSA program hubs

We reviewed materials published online by 4 other CTSA Program Hubs to identify and compare their data science platforms (compute environments, common data models, and user-facing tools) to the SDSR (Table 3). We found that the SDSR shares different design elements with the data science platforms developed and maintained by each of these hubs. The SDSR is most similar to UCSF’s data resources in its emphasis on self-serve data access and compute resource availability for researchers. UCSF also makes deidentified patient data available on-demand to researchers.^97^ In contrast, Harvard^98^ emphasizes services to support research, including bioinformatics and biostatistics consulting services, and its Streamlined, Multisite, Accelerated Resources for Trials (SMART) IRB Reliance Platform for creating, tracking and sharing study protocols, and streamlining IRB review. It also maintains research resources including RedCap, the ACT (Accrual to Clinical Trials) Network platform for querying patient data to assess feasibility for clinical studies across the CTSA Consortium, and the Harvard Catalyst Profiles resource for discovering research done by Harvard faculty. Duke, Harvard, and Vanderbilt use CDMs other than OMOP, while both Duke and Vanderbilt also provide user access to self-serve data search tools.

**Table 3.** Data science platforms at other CTSA Program Hubs

CTSA Program Hub	Compute environment(s)	Common data model(s)	User-facing tool(s)
Duke University^99–101^	Amazon Web Services; Google Cloud Platform; Microsoft Azure	PCORI	DEDUCE search tool
Harvard University^102^^,^^103^	Amazon Web Services; Google Cloud Platform; Microsoft Azure; PACE private cloud services	i2b2	Harvard Catalyst Profiles, SHRINE network query tool, i2b2 tranSMART, REDCap, SMART IRB Reliance Platform
Vanderbilt University^104^^,^^105^	Microsoft Azure; Google Cloud Platform; VUMC private cloud services	OMOP, PCORI, i2b2	VUMC Office of Research Informatics Synthetic Derivative and Research Derivative search tools
University of California San Francisco^97^	Amazon Web Services; on-premise high-performance computing Linux environment	OMOP	PatientExploreR, JupyterHub, Hue SQL Assistant

## DISCUSSION

The primary goal of the SDSR is to make data assets available for research and provide computational resources to use those data. Just as libraries (which increasingly steward digital resources) are essential for schools and research institutions, patient data repositories are essentially “libraries” documenting the patient care experience, which is necessary for advancing the mission of AMCs. They are also essential for building a learning health system (LHS), as envisioned by the Institute of Medicine (IOM; now the National Academy of Medicine, or NAM), that leverages integrated digital infrastructure to provide data-driven and coordinated care centered on the patient. The NAM and National Science Foundation envision a LHS that can rapidly inform decisions and have transformative effects on health.^106^ An ecosystem such as the SDSR is necessary to provide exceptional care to patients and to inform health system evaluation and improvement with data.

The SDSR spans the School of Medicine, Stanford Health Care, and Stanford Children’s Health, which are legally separate entities and historically have had separate IT departments, each with its own Chief Information Officer. We retained an external consulting group, which advised us to unify the IT departments, and we embarked on that journey in 2017, completing it in 2021. TDS is the unified IT department for the School of Medicine and Stanford Health Care. As part of this unification, we removed redundancies such as separate Epic Clarity instances for operational and research use. Both the research data warehouse (STARR) and the operational enterprise data warehouse (Health Catalyst) are now populated from the same Clarity instance. The benefit of this separation-with-one-source is that we can provision deidentified data for research in a cloud hosted environment, enabling a high degree of researcher self-service^107^ with no impact on operational projects. The downside is that operational deployment of research innovations such as machine learning models for classification or prediction requires either retraining on operational data marts, or manual reconciliation of the features that are inputs to the model. Rapid (and ideally automated) reconciliation of the feature space between the research and operational data warehouses remains one of the biggest pain points in implementing machine learning models to guide care.^108^ So, while the separation has sped up research (ie, the creation of classifiers and predictors across a myriad of data types) their translation into improving care remains a bottleneck—leading to the creation of a new data science team in Stanford Health Care^109^ to streamline that process.

One potential limitation of SDSR’s design is the use of the OMOP CDM to structure clinical data in STARR. The ETL from Epic Clarity to the OMOP CDM requires many data elements from Clarity to be omitted from STARR, in order to conform to OMOP CDM rules and conventions. For example, medication record details such as directions for use (signetur) and frequency cannot be represented in the CDM’s drug exposure table. These data omissions limit the kinds of analyses that can be done using STARR data. The benefits of the ability to conduct network studies with other institutions using the OMOP CDM and the availability of community maintained statistical analysis packages available via OHDSI help to balance the cost of adhering to OMOP CDM design decisions.

Maintaining and improving the SDSR has also surfaced a number of challenges. First, SDSR open source tools and self-serve data access processes are free in the sense that they enable the least restrictive access possible, but they are not free in the sense of cost. The shift to cloud computing as the primary form of SDSR computational resources means that our costs have shifted from buying and managing hardware and hiring storage, server, database, and network administrators to paying for cloud services and hiring cloud DevOps engineers. In some areas, hiring software engineers who are experienced working with cloud services may be a challenge. Ensuring continued financial support for SDSR infrastructure and personnel via large center grants such as the CTSAs, internal institutional funding, and chargebacks to faculty research funds is a crucial process. Striking the right balance among these sources of financial support to ensure continued operation of the SDSR while maximizing researcher access remains a challenge, and one that is not unique to Stanford Medicine. Second, providing effective user training is essential. There is also a substantial cost trade-off in making tools “user friendly” versus training users to effectively use open source tools. Often, user training requires a larger upfront commitment, but training users is among the few scalable ways to create community learning that minimizes the need for project-specific, staff intensive concierge services. Last, finding the right ratio of tool builders and service providers to meet vastly differing user skill levels remains challenging. We currently maintain teams primarily of builders, with fewer service providers, as we emphasize user training and self-service access to data and analytic tools. We regularly solicit feedback from SDSR team members and users, which will provide insight as to the effects of this approach.

## CONCLUSION

Research patient data repositories and data science platforms are a cornerstone for research at AMCs and a prerequisite for LHSs. The SDSR ecosystem provides data, methods, tools, and personnel support for clinical and translational data science at Stanford Medicine with the goal of seeding a vibrant LHS. Our immediate efforts include providing data from diverse sources including EMRs, radiology, and cardiology imaging, bedside monitors, health system messaging services, wearables, and patient reported data, as well as tools that support a diverse set of downstream use cases and usage scenarios. We hope that our experiences and the design of the SDSR ecosystem will serve as an informative case study for teams at other AMCs. Keeping pace with the growing scale and complexity of modern health systems will be an ongoing challenge, but the need for an SDSR like ecosystem to support research, education, and innovation in clinical care is clear.

## Data Availability

The data underlying this article will be shared on reasonable request to the corresponding author.
